# Facile and Rapid Isolation of Oxypeucedanin Hydrate and Byakangelicin from *Angelica dahurica* by Using [Bmim]Tf_2_N Ionic Liquid

**DOI:** 10.3390/molecules26040830

**Published:** 2021-02-05

**Authors:** Alice Nguvoko Kiyonga, Gyeongmin Hong, Hyun Su Kim, Young-Ger Suh, Kiwon Jung

**Affiliations:** Institute of Pharmaceutical Sciences, College of Pharmacy, CHA University, Sungnam 13844, Korea; gabriella@chauniv.ac.kr (A.N.K.); gmini93@chauniv.ac.kr (G.H.); khs8812@snu.ac.kr (H.S.K.); ygsuh@cha.ac.kr (Y.-G.S.)

**Keywords:** ionic liquids, oxypeucedanin hydrate, byakangelicin, *A. dahurica*, back-extraction, enrichment

## Abstract

Ionic liquids (ILs) have sparked much interest as alternative solvents for plant materials as they provide distinctive properties. Therefore, in this study, the capacity of ILs to extract oxypeucedanin hydrate and byakangelicin from the roots of *Angelica dahurica* (*A. dahurica*) was investigated. The back-extraction method was examined to recover target components from the IL solution as well. Herein, [Bmim]Tf_2_N demonstrated outstanding performance for extracting oxypeucedanin hydrate and byakangelicin. Moreover, factors including solvent/solid ratio, extraction temperature and time were investigated and optimized using a statistical approach. Under optimum extraction conditions (solvent/solid ratio 8:1, temperature 60 °C and time 180 min), the yields of oxypeucedanin hydrate and byakangelicin were 98.06% and 99.52%, respectively. In addition, 0.01 N HCl showed the most significant ability to back-extract target components from the [Bmim]Tf_2_N solution. The total content of both oxypeucedanin hydrate (36.99%) and byakangelicin (45.12%) in the final product exceeded 80%. Based on the data, the proposed approach demonstrated satisfactory extraction ability, recovery and enrichment of target compounds in record time. Therefore, the developed approach is assumed essential to considerably reduce drawbacks encountered during the separation of oxypeucedanin hydrate and byakangelicin from the roots of *A. dahurica.*

## 1. Introduction

**Figure 1:**
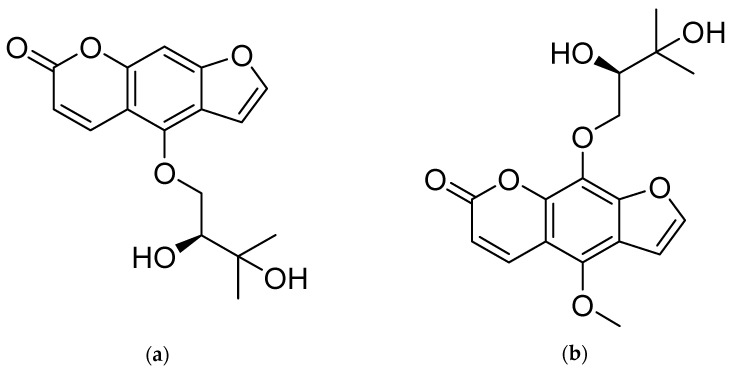
Chemical structures of (**a**) Oxypeucedanin hydrate, (**b**) Byakangelicin.

*Angelica dahurica* (Fisch. ex Hoffm.) Benth. et Hook. f. (*A. dahurica*) is a folk medicinal plant for which the roots have been used over the years as a remedy for cold, fever, headache, nasal congestion, toothache, asthma, stomachache and dysmenorrhea in East Asian countries (Korea, China, Russia, Taiwan, etc.) [1,2,3,4]. Several previous studies have also reported that the roots of *A. dahurica* exhibit various pharmacological functions, including antibacterial, anti-asthmatic, hypotensive, anti-inflammatory, antioxidation, anti-cancer, and anti-Alzheimer effects [5,6,7,8,9,10,11]. Pharmacological functions of *A. dahurica* are generally attributed to coumarins (oxypeucedanin, bergapten, imperatorin, cnidilin, isoimperatorin, xanthotoxol, oxypeucedanin hydrate, byakangelicin, etc.), which are major constituents of this herb [12,13,14]. Oxypeucedanin hydrate (Figure 1a) has inhibitory effects on tyrosinase activity [15] and exhibits anti-inflammatory and antioxidant activities [16]. Byakangelicin (Figure 1b) has been reported to modulate the brain distribution of various bioactive compounds by improving accumulation and consequently boosting their curative effectiveness [17]. Byakangelicin shows effect against carbon tetrachloride-induced liver injury and fibrosis in mice as well [18].

Ionic liquids (ILs) are a broad class of molten salts entirely composed of ions, and they possess a melting point below 100 °C [19,20]. ILs possess distinctive properties such as great selectivity and electric conductivity, negligible vapor pressure and low volatility [21], poor flammability [22], strong thermal stability [23], and solvation power of organic [24] and inorganic [25] compounds. ILs are also referred to as “designer” solvents because their physicochemical characteristics can be tuned by altering their cations or anions [26]. The remarkable properties of ILs have engendered enormous interest among scientists in diverse fields, enabling them to be considered as green media and a promising substitute for volatile organic solvents (VOCs). Therefore, ILs have been applied in diverse fields such as biochemistry [27], electrochemistry [28], analytical chemistry [29], pharmaceutics [30], medicine [31], and so forth. In analytical chemistry, ILs have been applied for the extraction and separation of several bioactive components from herbal products, namely, phenolics [32], alkaloids [33,34], flavonoids [35,36], ginsenosides [37], coumarins [38], terpenoids [39,40], and so forth [41,42].

Back-extraction, also called liquid-liquid extraction, is a purification process in which a compound or a mixture of compounds transfer from one phase to another based on their differential solubility. In this process, two essentially immiscible or partially soluble solvents are utilized to allow compound transfer and separation. The back-extraction process is routinely employed by chemists due to numerous advantages, such as simplicity, low cost, ease of scale up and rapidity [43]. In their study, Absalan and co-workers successfully employed [BMIm]PF_6_ for extracting 3-indole-butyric acid from its aqueous concentrate and effectively back-extracted the compound from the IL phase [44]. Moreover, Larriba and co-workers were able to back-extract tyrosol from the ILs using 0.1M NaOH aqueous solution as well [45].

The separation of active components in general and particularly of minor compounds from plant raw materials is exhausting and tedious. In that regard, this study aimed to develop an effective and relevant approach for the extraction and enrichment of minor coumarins, oxypeucedanin hydrate and byakangelicin, from the roots of *A dahurica* using ILs as the extraction solvent and the back-extraction method for their recovery. The ultimate purpose was to overcome drawbacks of traditional separation techniques. Conventional separation methods are exhausting and time-consuming because they involve repetitive chromatographic steps. However, in this study, IL was effectively utilized as an extraction solvent while the back-extraction method was satisfactorily employed for the enrichment of target molecules from the IL solutions. To improve the rates of extraction and recovery of two compounds, several parameters (solvent/solid ratio, extraction temperature and extraction time) were studied and optimized by response surface methodology (RSM). As far as we know, this is the first time a procedure using ILs and back-extraction technique has been consistently achieved for the separation and enrichment of oxypeucedanin hydrate and byakangelicin from the roots of *A. dahurica*. This study also confirmed the ability of ILs as separation media for active substances of plant origin.

## 2. Results and Discussion

### 2.1. Screening of ILs

**Figure 2:**
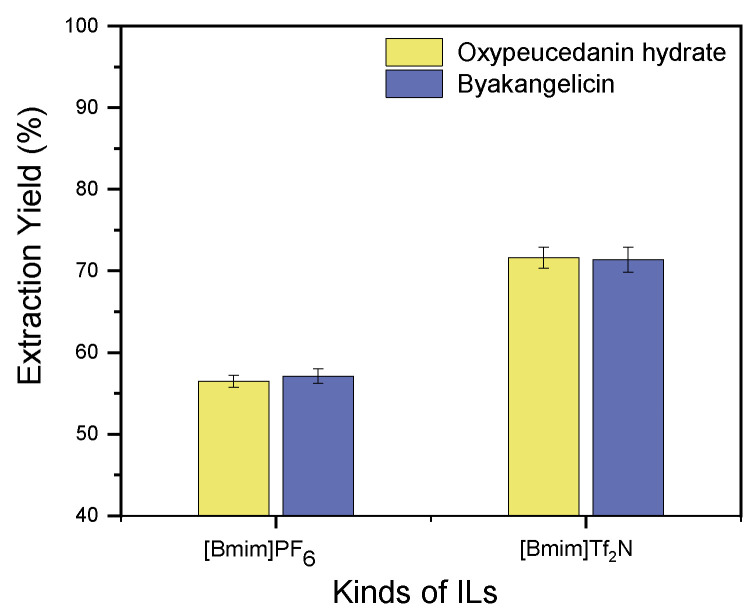
Effect of ionic liquids (ILs) on the extraction of oxypeucedanin hydrate and byakangelicin.

Two imidazolium-based hydrophobic ILs, [Bmim]PF_6_ and [Bmim]Tf_2_N, were evaluated and compared for their ability to extract oxypeucedanin hydrate and byakangelicin from the roots of *A. dahurica*. These two ILs were selected in this study due to their easy availability in the market and moderate cost compared to commonly employed room temperature ILs. For the extraction experiments, powdered samples (one gram each) were extracted with IL at a 6:1 solvent/solid ratio and 40 °C extraction temperature for 120 min. The agitation speed was 500 rpm. Note that samples were soaked for 30 min prior to their extraction. The results revealed that [Bmim]Tf_2_N demonstrated the highest extraction performance for oxypeucedanin hydrate and byakangelicin, 71.64% (872.996 μg)) and 71.38% (1074.78 μg)), respectively. Extraction yields of oxypeucedanin hydrate and byakangelicin obtained from (Bmim)PF_6_ were, respectively, 56.48% (688.22 μg)) and 57.11% (859.98 μg)) (Figure 2). Based on the principle “like dissolves like”, it was assumed that target components were extracted with excellent yields in [Bmim]Tf_2_N because they possess similar polarity with [Bmim]Tf_2_N than with [Bmim]PF_6_. On the other hand, the difference in viscosity between [Bmim]Tf_2_N and [Bmim]PF_6_ [46,47] was assumed to have impacted the extraction abilities of these ILs. Scientists have suggested that an increase in viscosity hinders the imbibement of the solvent into the plant tissues, which therefore has a negative impact on the mass transfer of compounds [48,49]. In this regard, the closeness of [Bmim]Tf_2_N polarity with that of the target components and its low viscosity compared to [Bmim]PF_6_ were presumed as the driving forces leading to the improvement of oxypeucedanin hydrate and byakangelicin extraction yields. Because [Bmim]Tf_2_N showed the highest extraction efficiency for both target components, it was selected for further experiments.

### 2.2. Single Factor Experiments

#### 2.2.1. Effect of the Solvent/Solid Ratio on the Extraction of Oxypeucedanin Hydrate and Byakangelicin

**Figure 3:**
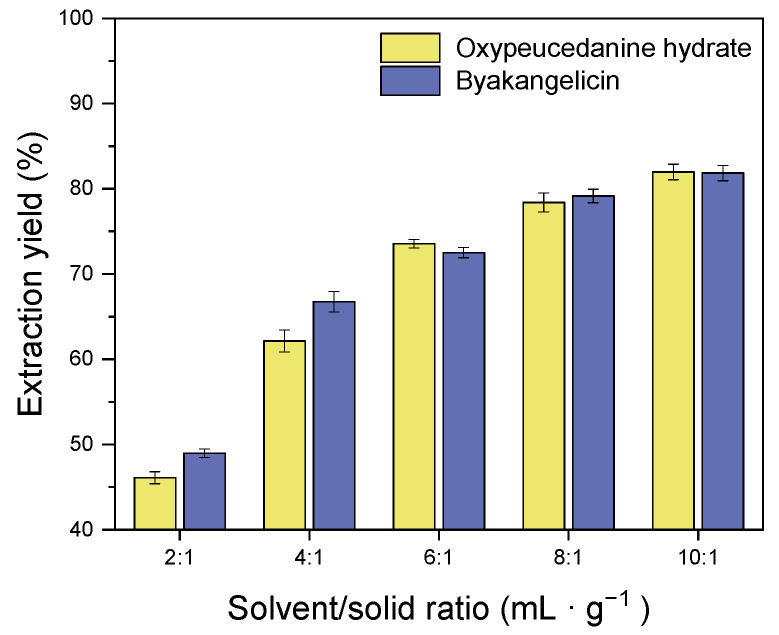
Effect of solvent/solid ratio on the extraction of oxypeucedanin hydrate and byakangelicin.

The extraction process was carried out at 2:1, 4:1, 6:1, 8:1 and 10:1 solvent/solid ratios to examine their effect on the extraction of oxypeucedanin hydrate and byakangelicin. This was in the aim of improving the extraction of both compounds while preventing incomplete extraction and waste of the solvent. Herein, the extraction temperature and time were set as 40 °C and 120 min, respectively, and the agitation speed was 500 rpm. Note that the samples were soaked for 30 min prior to their extraction. From the results, the extraction of oxypeucedanin hydrate and byakangelicin increased with the increment of the solvent/solid ratio. In addition, the most significant extraction of approximately 81% was achieved when the ratio was 10:1 (Figure 3).

#### 2.2.2. Effect of Temperature on the Extraction of Oxypeucedanin Hydrate and Byakangelicin

**Figure 4:**
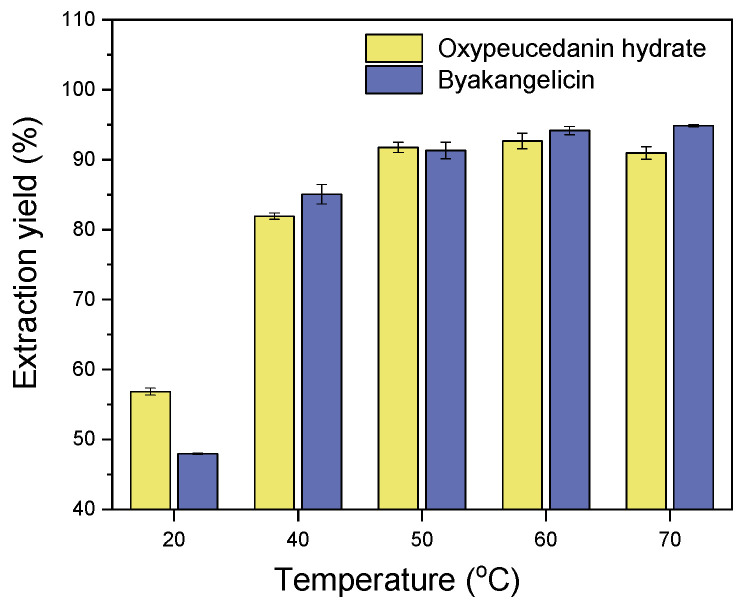
Effect of temperature on the extraction of oxypeucedanin hydrate and byakangelicin.

Temperature is one of several factors investigated during the extraction process as it is known for either positively (by improving the extraction) or negatively (by causing the degradation of heat-sensible components) impacting the extraction of active components. In this regard, experiments were performed to investigate the impact of various extraction temperatures (20 °C, 40 °C, 50 °C, 60 °C, and 70 °C) on the extraction of oxypeucedanin hydrate and byakangelicin. The solvent/solid ratio was maintained at 10:1, the extraction time at 120 min and the agitation speed at 500 rpm. Note that the samples were soaked for 30 min prior to their extraction. As shown in Figure 4, the extraction of oxypeucedanin hydrate and byakangelicin greatly improved when the temperature increased from 20 °C to 60 °C. At 60 °C, the extraction yields of oxypeucedanin hydrate and byakangelicin were 92.67% and 94.16%, respectively. Thus, 60 °C was selected for further experiments.

#### 2.2.3. Effect of Time on the Extraction of Oxypeucedanin Hydrate and Byakangelicin

**Figure 5:**
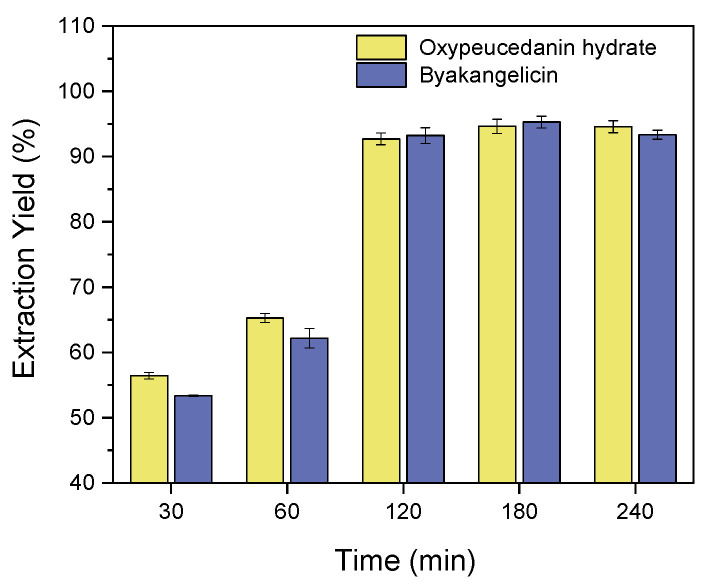
Effect of time on the extraction of oxypeucedanin hydrate and byakangelicin.

The effect of time on the extraction of oxypeucedanin hydrate and byakangelicin was studied for improving the extraction of both compounds while preventing incomplete extraction and time-waste. The time parameter was varied as follows: 30, 60, 120, 180 and 240 min. Other conditions were: extraction temperature 60 °C, solvent/solid ratio 10:1 and agitation speed 500 rpm. Note that the samples were soaked for 30 min prior to their extraction. As depicted in Figure 5, the most notable extraction of two compounds was observed when the extraction time was 180 min. At this time, the extraction efficiency of oxypeucedanin hydrate and byakangelicin reached 94.64% and 95.29%, respectively.

### 2.3. Statistical Optimization of the Extraction Conditions of Oxypeucedanin Hydrate and Byakangelicin

For the statistical optimization of the extraction of oxypeucedanin hydrate and byakangelicin from the roots of *A. dahurica*, three variables including solvent/solid ratio, extraction temperature, and extraction time were optimized using RSM. The conditions for extraction variables were established based on data obtained in Section 2.2 and are illustrated in Table 1 and Table 2 along with corresponding experimental data. X_1_ is a code for the extraction time variable, X_2_ is the extraction temperature variable and X_3_ is the solvent/solid ratio variable.

The analysis of variance for response surface quadratic models is summarized in Table 3. The *p*-values (*p* <0.05) and the regression coefficient (R^2^ >0.95) were used to assess the significance of the model. The regression coefficients (R^2^ = 0.989 for oxypeucedanin hydrate and R^2^ = 0.987 for byakangelicin) indicated that only 1.0% of the variations were not explained by the model and that the model had high reliability (Table 3).

As seen in Table 3, sole linear term coefficients X_1,_ X_2,_ and X_3_ were significant with *p*-values inferior to 0.05. This confirmed that a linear model was adequate to represent our model. The “lack of fit”for *p*-values (*p* = 0.23 for oxypeucedanin hydrate and *p* = 0.40 for byakangelicin) and the “lack of fit” for F-values (F = 3.58 for oxypeucedanin hydrate and F = 1.67 for byakangelicin) indicated that the model was significant and suitable for obtaining optimum extraction of oxypeucedanin hydrate and byakangelicin (Table 3). The second-order differential equations for obtaining oxypeucedanin hydrate and byakangelicin are illustrated below.

Yield of oxypeucedanin hydrate = 87.3076 + 7.944X_1_ + 4.0578X_2_ + 4.7509X_3_ − 0.4253X_1_^2^ + 0.2437X_2_^2^ + 1.6173X_3_^2^ + 0.1489X_1_X_2_ + 0.2199X_1_X_3_ − 0.3581 X_2_X_3_ (1)

Yield of byakangelicin = 89.4976 + 6.4364X_1_ + 4.8599X_2_ + 5.8638X_3_ + 0.6384X_1_^2^ − 0.2631X_2_^2^ − 1.0769X_3_^2^ − 1.0640X_1_X_2_ + 0.2363X_1_X_3_ − 0.3168X_2_X_3_ (2)

**Figure 6:**
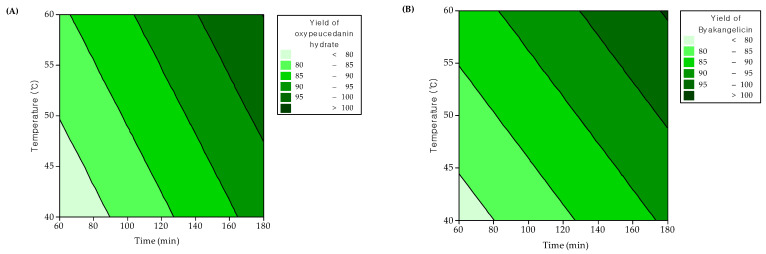

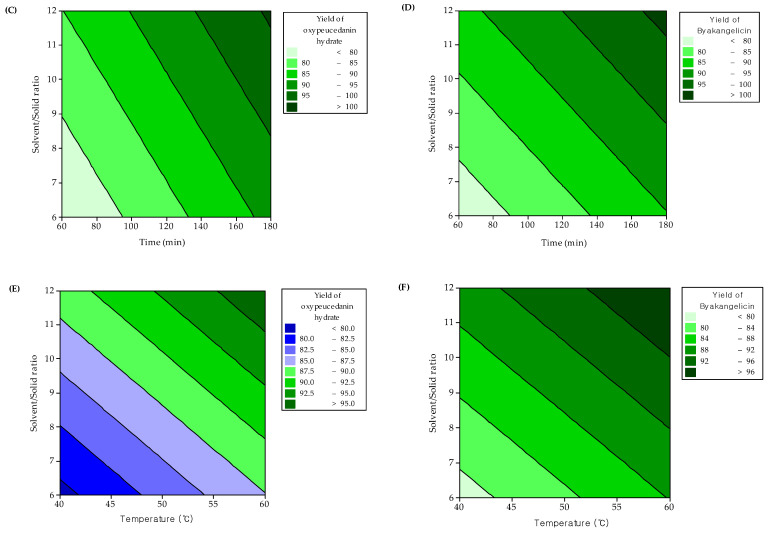
Contour plots of extraction yields of oxypeucedanin hydrate and byakangelicin versus variables. (**A**) effect of temperature and time on extraction yield of oxypeucedanin hydrate, (**B**) effect of temperature and time on extraction yield of byakangelicin, (**C**) effect of solvent/solid ratio and time on extraction yield of oxypeucedanin hydrate, (**D**) effect of solvent/solid ratio and time on extraction yield of byakangelicin, (**E**) effect of solvent/solid ratio and temperature on extraction yield of oxypeucedanin hydrate and (**F**) effect of solvent/solid ratio and temperature on extraction yield of byakangelicin.

In Figure 6, the response surface plots indicating the interaction between parameters and their effects on the extraction of oxypeucedanin hydrate and byakangelicin are displayed. The solvent/solid ratio of 8:1 at an extraction temperature of 60 °C for 180 min were the optimum conditions proposed by RSM to improve the extraction yields of oxypeucedanin hydrate and byakangelicin up to 97.98% and 98.01%, respectively.

### 2.4. Extraction of Oxypeucedanin Hydrate and Byakangelicin Using Optimal Extraction Conditions

Samples were extracted under optimum extraction conditions proposed by RSM for examining the precision and validity of the method. The proposed optimum conditions were as follows: solvent/solid ratio 8:1, extraction temperature 60 °C and extraction time 180 min. The results predicted by RSM and the experimental results are depicted in Table 4. As observed in Table 4, the experimental values agreed with the predicted values. Accordingly, it is presumed that the RSM model is appropriate to estimate the extraction of oxypeucedanin hydrate and byakangelicin from the roots of *A. dahurica*.

### 2.5. Isolation of Oxypeucedanin Hydrate and Byakangelicin from the IL Solution Using Back-Extraction

The back-extraction process was conducted for the isolation and enrichment of oxypeucedanin hydrate and byakangelicin from the [Bmim]Tf_2_N extraction solution. Preliminary investigation was conducted using deionized water (DIW) and hexane as back-extraction solvents because of their capability to form a two-phase system with the [Bmim]Tf_2_N. From the preliminary experiment, it was found out that DIW exhibited promising isolation performance compared to hexane, as DIW showed high selectivity in recovering oxypeucedanin hydrate and byakangelicin. However, using hexane as a back-extraction solvent led to unsatisfactory results as several components present in the [Bmim]Tf_2_N solution were simultaneously back-extracted in the hexane phase. This was presumably due to the fact that most components of the [Bmim]Tf_2_N solution migrated to the hexane phase as they were more highly soluble in hexane than in [Bmim]Tf_2_N.

Because DIW demonstrates promising outcomes, various aqueous solutions (DIW, 0.01 N NaOH, and 0.01 N HCl) were evaluated for improving the rate of recovery and for the enrichment of target components in the final product. The initial amounts of oxypeucedanin hydrate and byakangelicin in the [Bmim]Tf_2_N solution were 609.90 μg and 729.81 ug, respectively. The volume of [Bmim]Tf_2_N solution was 4 mL and the volume of the back-extraction solvent was set as 30 mL (1:7.5 IL/back-extraction solvent). The equilibration time was fixed for 4 h to allow complete phase-equilibration. From the results (Table 5), when 0.01 NaOH was used as a back-extraction solvent, no target compound could be recovered. However, when DIW and 0.01 N HCl were employed, oxypeucedanin hydrate and byakangelicin could be recovered from the [Bmim]Tf_2_N extraction solution. Nevertheless, the most outstanding performance was acquired when 0.01 N HCl was utilized (Table 5).

In addition, various volumes (10, 20, 30, 40 and 50 mL) of 0.01 N HCl back-extraction solvent were investigated to improve the rate of recovery of oxypeucedanin hydrate and byakangelicin. The volume of [Bmim]Tf_2_N solution was maintained as 4 mL. Namely, the ratios of IL to back-extraction solvent were as follows: 1:2.5, 1:5, 1:7.5, 1:10, and 1:12.5. The initial amounts of oxypeucedanin hydrate and byakangelicin in the [Bmim]Tf_2_N solution, the volume of [Bmim]Tf_2_N solution and the equilibration time were as above. As the results, 40 mL (1:10 IL/back-extraction solvent) was the adequate volume of 0.01 N HCl back-extraction solvent as it exhibited satisfactory recovery yields of both oxypeucedanin hydrate and byakangelicin: 91.99% (561.06 μg) and 89.17% (650.76 μg), respetively (Table 6). This result can be explained by the fact that, when using 0.01 N HCl as back-extraction medium, ion exchange occurred between Cl and Tf_2_N anions because the interaction energy between the Bmim cation and Cl anion is relatively greater than between the Bmim cation and Tf_2_N [50]. However, due to the presence of a low concentration of Cl anion in the formed system, it is assumed that both [Bmim]Tf_2_N and [Bmim]Cl coexisted in the IL phase. The change in the IL solution composition is believed to have influenced the decrease in oxypeucedanin hydrate and byakangelicin solubilities in the IL phase. Consequently, this change has favorized their migration to the aqueous phase. Nevertheless, oxypeucedanin hydrate and byakangelicin possessed inferior solubilities in the back-extraction solution. Therefore, as observed in Table 6, their recovery amounts were enhanced with the increase in the amount of the back-extraction solvent.

Several laboratory (researching) experiments are carried out at small-scale using small volume sizes of materials. However, the moving of promising experiments from small-scale to large-scale is challenging and sometimes unsuccessful due to some limitations encountered. Therefore, in this study, the back-extraction experiment was carried out at a larger scale to confirm whether the results agreed with data obtained at small-scale. The contents (purities) of oxypeucedanin hydrate and byakangelicin in the final product were investigated as well. The initial amounts of oxypeucedanin hydrate and byakangelicin in the [Bmim]Tf_2_N solution were 5930.02 and 7370.23 μg, respectively. The volume of the back-extraction solvent was 400 mL (1:10 IL/back-extraction solvent), and the equilibration time was fixed for 4 h. As illustrated in Table 7, both oxypeucedanin hydrate and byakangelicin were obtained in satisfactory yield (approximately 90%), and these results agreed with data obtained from small-scale experiments (Table 6). Moreover, the total purity of both components exceeded 80% in the final product, namely, 36.99% oxypeucedanin hydrate and 45.12% byakangelicin.

**Figure 7:**
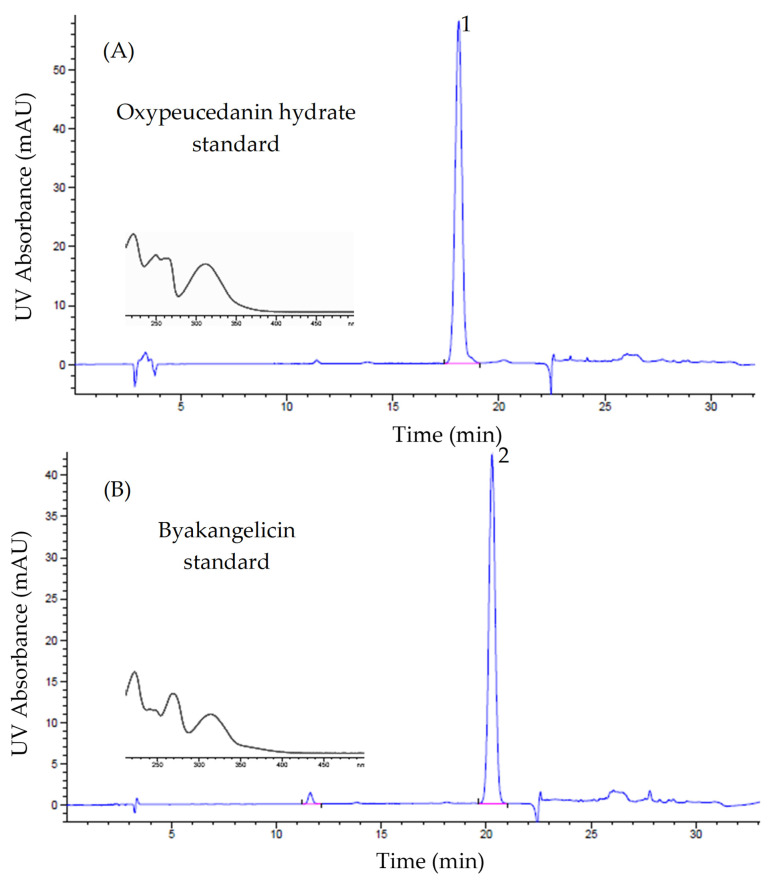

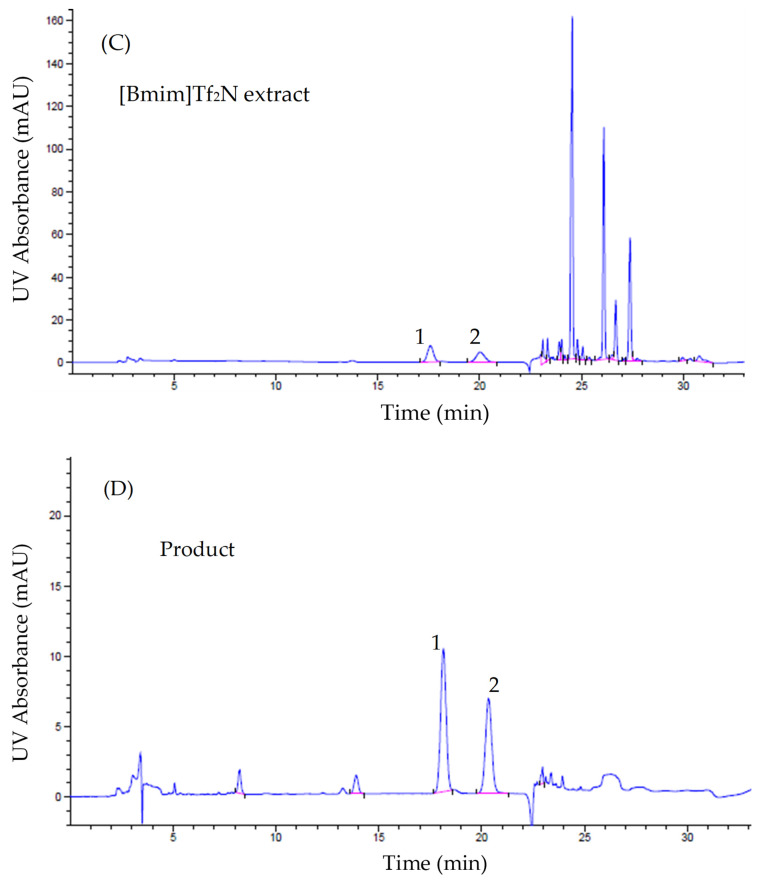
HPLC chromatograms of (**A**) oxypeucedanin hydrate standard, (**B**) byakangelicin standard, (**C**) [Bmim]Tf_2_N extraction solution and (**D**) product. (254 nm).

From this experiment, the enrichment of both oxypeucedanin hydrate and byakangelicin, two minor coumarins of the roots of *A. dahurica*, was achieved in a short period and few steps. This is assumed to considerably reduce the time and effort needed for the separation of these two components. HPLC chromatograms of oxypeucedanin hydrate and byakangelicin standards, [Bmim]Tf_2_N extraction solution and product are illustrated in Figure 7.

### 2.6. Comparison of the Extraction and Separation Method of Oxypeucedanin Hydrate and Byakangelicin

The roots of *A. dahurica* were extracted with DIW and 50 and 95% ethanol to compare the ability of these frequently employed extraction solvents with that of IL. These solvents were specifically extracting oxypeucedanin hydrate and byakangelicin. Experiments were performed at the same extraction conditions (solvent/solid ratio 8:1, extraction temperature 60 °C and extraction time 180 min) proposed by RSM when IL was employed as the extraction solvent. The results are illustrated in Table 8. As observed, at the same extraction conditions, the IL [Bmim]Tf_2_N exhibited the highest extraction ability for oxypeucedanin hydrate (98.06%) and byakangelicin (99.52%) compared to other solvents (Table 8).

In addition, efficient, facile and rapid isolation and enrichment techniques of oxypeucedanin hydrate and byakangelicin from *A. dahurica* using the above-mentioned solvents have yet been unreported in the literature. This is assumed to be because of the laborious and exhausting separation techniques of active components from plant raw materials. More importantly, this requires expensive equipment and/or repetitive chromatography. Moreover, the process is demanding, especially when it comes to the separation of minor compounds such as oxypeucedanin hydrate and byakangelicin. However, a facile and rapid method for the separation (enrichment) of minor coumarins, oxypeucedanin hydrate and byakangelicin, from *A. dahurica* using IL as the extraction solvent was successfully achieved. The process was completed in few steps and within record time compared to traditional techniques that require repetitive chromatography. The novel developed approach is believed to considerably reduce the steps and time required for the isolation of oxypeucedanin hydrate and byakangelicin from the roots of *A. dahurica*. Therefore, the developed approach is assumed to overcome the drawbacks of traditional methods by preventing time wasting and by lessening labor cost.

## 3. Materials and Methods

### 3.1. Reagents and Materials

*A. dahurica* was purchased from an oriental herbal medicine market in Seoul, Korea, in 2019. Authentication of the plant material was conducted by one of the authors, Professor Kiwon Jung (College of Pharmacy, CHA University, Pocheon-si, Korea). Moreover, voucher specimen (HPC-AR01) was archived in the Herbarium of the College of Pharmacy, CHA University. Oxypeucedanin hydrate and byakangelicin standards (HPLC purity > 95%) were directly isolated from *A. dahurica*. From A-star Co., Ltd. (Ulsan, Korea), 1-Butyl-3-methylimidazolium bis(trifluoromethylsulfonyl) imide [Bmim]Tf_2_N and 1-Butyl-3-methylimidazolium hexafluorophosphate [Bmim]PF_6_ ILs were purchased. HPLC grade deionized water (DIW) and acetonitrile and analytical grade ethanol (purity > 98%), hexane and hydrochloric acid were all purchased from Daejung Chemicals (Siheung-si, Korea).

### 3.2. Methods

#### 3.2.1. Extraction Procedure and Quantitative Evaluation of Oxypeucedanin Hydrate and Byakangelicin in the IL Extraction Solutions

Samples of one gram (1.0 g) of grounded *A. dahurica* roots were mixed with known volumes of two hydrophobic ILs, [Bmim]Tf_2_N and [Bmim]PF_6_, and soaked for 30 min at constant rotational speed (500 rpm). The samples were then extracted on a hot plate at 40 °C for 120 min. The purpose was to evaluate the extraction ability of two ILs on the extraction of oxypeucedanin hydrate and byakangelicin, thus selecting the promising IL for further experiments. After selecting the relevant IL, experiments were carried out on the selected IL. Then, the solid/solvent ratio, extraction temperature and time were cautiously investigated, and RSM employing Box–Behnken design was performed to optimize the extraction yields of target compounds. Minitab 16 was the software. After each extraction, samples were vacuum-filtered through 0.45 μm membrane filters and 50 μL of each filtrate was diluted with acetonitrile to make 1 mL solution. Prepared sample solutions were filtered onto 0.45 μm nylon membranes and then analyzed by HPLC to determine the extraction yield. All experiments were conducted in duplicate and average values were reported. The following equation was employed to determine the extraction efficiency:

(3) $$\mathrm{Extraction}\;\mathrm{yield}\;\left(\%\right)\;=\frac{M_{1}\left(W\right)}{M\;\left(W\right)}\;\times 100$$

where M_1_ is the content of target compound in the extract, M is the content of the target compound in the plant material and W is the code for weight.

#### 3.2.2. High-Performance Liquid Chromatography (HPLC) Analysis

HPLC measurements were performed on an Agilent 1260 Infinity Ⅱ LC system (CA, USA) equipped with a G7111A quaternary gradient pump, a G7129C autosampler and a G7115A UV-detector. Compounds were separated in an Aegispak C18-L (5 μm, 4.6 mm × 250 mm, YoungJin Biochrom, Seongnam-si, Korea) analytical column at a flow rate of 1.0 mL/min and a column temperature of 30 °C. The injection volume was 10 μL and the UV detection wavelength was set at 254 nm. The mobile phases consisted of 0.1% phosphoric acid-water (A) and acetonitrile (B). The time program of the gradient elution of acetonitrile was as following: 25–30% (0–18 min), 30–70% (18–20 min), 70% (20–28 min), then followed by a return to the initial conditions 70–25% (28–29 min) and kept 6 min (29–35 min) for the column equilibrium.

The chromatographic plots for oxypeucedanin hydrate and byakangelicin constructed on three consecutive runs showed good linearities (correlation coefficient, R^2^ = 0.999) within the investigated range (2.5 ug–60 μg·mL^−1^). Y = 33.18x − 29.51 was the regression equation for oxypeucedanin hydrate while Y = 18.79x − 5.76 was the regression equation for determining byakangelicin. The method also showed good accuracies (mean recoveries: 97.85% and 99.28%) and precisions (RSD%: 3.63 and 2.67) for both oxypeucedanin hydrate and byakangelicin, respectively. As follows, the above HPLC method was confirmed adequate for the quantitative determination of oxypeucedanin hydrate and byakangelicin in further analyses.

#### 3.2.3. Quantitative Evaluation of Oxypeucedanin Hydrate and Byakangelicin

The purpose of this experiment was to determine the total amount of oxypeucedanin hydrate and byakangelicin in the roots of *A. dahurica.* Herein, three different samples of one gram each were extracted with 50 mL of 95% ethanol at room temperature. The extraction process was repeated until oxypeucedanin hydrate and byakangelicin were undetected anymore in the extraction solution. Afterwards, extraction solutions of each sample were separately collected, concentrated under reduced pressure, and further analyzed by HPLC. The average amounts of oxypeucedanin hydrate and byakangelicin in the extract were 1218.50 μg (0.12% in the raw material) and 1505.75 μg (0.15% in the raw material), respectively. The above contents were considered as reference amounts of oxypeucedanin hydrate and byakangelicin in the roots of *A. dahurica* and were used for comparative studies.

#### 3.2.4. Isolation of Oxypeucedanin Hydrate and Byakangelicin from the IL Extraction Solution

The isolation (recovery) of two minor coumarins, oxypeucedanin hydrate and byakangelicin, from the IL extraction solution was performed using the back-extraction method. DIW and hexane were selected for this purpose because of their unique characteristic to form a two-phase system with the employed IL. The ability of each solvent for recovering oxypeucedanin hydrate and byakangelicin from the IL extraction solution was investigated. The separation process was conducted in a separation funnel by adding a known amount of back-extraction solvent and IL extraction solution to the funnel. The funnel was vigorously shaken and then the system was maintained to equilibrate at room temperature. After equilibration, the upper phase was separated from the lower phase. Subsequently, the upper phase was concentrated using rotary evaporator and then dried in vacuum overnight. Moreover, obtained extracts were analyzed by HPLC to determine the rate of recovery of target components. Further, the procedure was optimized to improve the rate of recovery and the contents (purity) of oxypeucedanin hydrate and byakangelicin in the final product. The following equations were utilized to measure the rate of recovery and the contents of both components in the product:

(4) $$\mathrm{Recovery}\;\left(\%\right)\;=\frac{M_{2}\left(W\right)}{M_{1}\;\left(W\right)}\times 100$$

(5) $$\mathrm{Content}\;\left(\%\right)\;=\frac{M_{2}\left(W\right)}{M_{4}\;\left(W\right)}\;\times 100$$

where M_1_, M, and W are the same as in Equation (3). M_2_ is the content of target compound in the product and M_4_ represents the total amount of the product.

#### 3.2.5. Structural Identification

Solution-state ^1^H-NMR and ^13^C-NMR analyses were performed, respectively, in CDCl_3_ on 800 MHz and 200 MHz NMR spectrometers (Advance, Bruker, Billerica, MA, USA). Precisely, these analyses confirmed the chemical structures of oxypeucedanin hydrate and byakangelicin reference standards isolated from the *A. dahurica*. Obtained data were compared with those reported in the literature [51].

Oxypeucedanin hydrate ^1^H-NMR and ^13^C-NMR spectra were identical with those reported in the literature [51]. ^1^H-NMR (CDCl3, 800 MHz) δ 8.14 (d, *J* = 9.7 Hz, 1H), 7.58 (d, *J* = 2.3 Hz, 1H), 7.13 (s, 1H), 6.97 (dd, *J* = 2.3, 0.8 Hz, 1H), 6.25 (d, *J* = 9.7 Hz, 1H), 4.52 (dd, *J* = 9.7, 2.9 Hz, 1H), 4.42 (dd, *J* = 9.7, 7.9 Hz, 1H), 3.89 (dd, *J* = 7.8, 2.8 Hz, 1H) 2.94 (bs, 1H), 2.24 (bs, 1H), 1.34 (s, 3H), 1.29 (s, 3H); ^13^C-NMR (CDCl3, 200 MHz) δ 161.1, 158.1, 152.5, 148.5, 145.3, 139.0, 114.2, 113.0, 107.3, 104.7, 94.8, 76.5, 74.4, 71.6, 26.7, 25.1.

Byakangelicin ^1^H-NMR and ^13^C-NMR spectra were identical with those reported in the literature [51]. ^1^H-NMR (CDCl3, 800 MHz) δ 8.10 (d, *J* = 9.8 Hz, 1H), 7.62 (d, *J* = 2.3 Hz, 1H), 7.00 (d, *J* = 2.3 Hz, 1H), 6.27 (d, *J* = 9.8 Hz, 1H), 4.58 (dd, *J* = 10.2, 2.6 Hz, 1H), 4.25 (dd, *J* = 10.2, 7.9 Hz, 1H), 4.17 (s, 3H), 3.81 (dd, *J* = 7.9, 2.6 Hz, 1H), 1.30 (s, 3H), 1.26 (s, 3H); ^13^C-NMR (CDCl3, 200 MHz) δ 160.1, 150.2, 145.2, 144.9, 144.0, 139.4, 126.8, 114.5, 112.9, 107.5, 105.3, 76.1, 75.9, 71.5, 60.7, 26.7, 25.0.

## 4. Conclusions

In this study, a methodology for the enrichment of oxypeucedanin hydrate and byakangelicin from *A. dahurica* has been successfully developed. Herein, IL was the extraction solvent and the back-extraction method was used for both components from the IL solution. Several conditions were studied and optimized. Under optimum separation conditions, the developed approach demonstrated satisfactory extraction efficiency, recovery and enrichment of both components. The extraction and recovery yields were, respectively, 98.06% and 91.99% for oxypeucedanin hydrate, and 99.52% and 89.17% for byakangelicin. The contents of oxypeucedanin hydrate and byakangelicin were 36.99% and 45.12%, respectively. Namely, the total content of both components reached up to 80% in the product. Generally, the separation of minor compounds from plant raw materials is a tedious and time-consuming task which entails repetitive chromatographic experiments. However, employing the proposed method, the enrichment of minor coumarins, oxypeucedanin hydrate and byakangelicin, from *A. dahurica* was achieved in few steps. The novel developed approach considerably reduces the steps and time for isolating oxypeucedanin hydrate and byakangelicin from the roots of *A. dahurica*. Therefore, this approach alleviates the drawbacks of traditional methods. This study also proved the potentiality of IL as an effective solvent for the separation of bioactive components from plant materials. The facile and high accessibility to minor active components is assumed to boost research related to these compounds. Consequently, this inevitably increases their potential application as lead compounds and/or drug candidates in the pharmaceutical industry.

## Figures and Tables

**Table 1:** Three level factorials for optimization of oxypeucedanin hydrate and byakangelicin extraction.

Levels	X_1_ Extraction Time (min)	X_2_ Extraction Temperature (°C)	X_3_ Solvent/Solid Ratio (mL·g^−1^)
Low level (−1)	60	40	6:1
Average (0)	120	50	9:1
High level (1)	180	60	12:1

**Table 2:** Box–Behnken design and response values for optimization of oxypeucedanin hydrate and byakangelicin extraction.

Run	X_1_ Extraction Time (min)	X_2_ Extraction Temperature (°C)	X_3_ Solvent/Solid Ratio (mL·g^−1^)	Extraction Yield of Oxypeucedanin Hydrate (%)	Extraction Yield of Byakangelicin (%)
1	120	50	9:1	87.55	88.54
2	120	50	9:1	88.01	89.07
3	180	60	9:1	100.20	101.19
4	180	50	6:1	90.74	89.56
5	120	60	12:1	97.88	99.06
6	60	50	6:1	77.21	77.94
7	120	50	9:1	86.36	90.87
8	120	60	6:1	88.64	85.59
9	60	50	12:1	85.82	88.09
10	180	40	9:1	91.86	92.22
11	60	60	9:1	82.10	89.66
12	180	50	12:1	100.23	100.65
13	120	40	6:1	79.74	77.89
14	60	40	9:1	74.35	76.43
15	120	40	12:1	90.41	90.09

**Table 3:** Variance analysis for the quadratic response surface regression model.

	Degrees of Freedom	Sum of Squares	Mean Square	*F*-Value	*p*-Value	R^2^	R^2^ Adjusted
Extraction yield of oxypeucedanin hydrate (%)							
Model	9	828.83	92.09	49.90	0.00		
X_1_	1	504.87	504.87	147.58	0.00		
X_2_	1	131.72	131.725	71.37	0.00		
X_3_	1	180.57	180.57	97.83	0.00		
X_1_^2^	1	1.16	0.67	0.36	0.57		
X_2_^2^	1	0.05	0.22	0.12	0.74	0.989	0.969
X_3_^2^	1	9.66	9.66	5.23	0.07		
X_1_ X_2_	1	0.09	0.09	0.05	0.83		
X_1_ X_3_	1	0.19	0.19	0.10	0.76		
X_2_ X_3_	1	0.51	0.51	0.28	0.62		
Residual	5	9.23	1.85				
Lack of fit	3	7.78	2.59	3.58	0.23		
Pure error	2	1.45	0.724				
Total	14	838.06					
Extraction yield of byakangelicin (%)							
Model	9	807.01	89.67	42.82	0.00		
X_1_	1	331.42	331.42	158.25	0.00		
X_2_	1	188.95	188.95	90.22	0.00		
X_3_	1	275.08	275.08	131.35	0.00		
X_1_	1	2.01	1.50	0.72	0.43		
X_2_^2^	1	0.12	0.26	0.12	0.74		
X_3_^2^	1	4.28	4.28	2.04	0.21	0.987	0.964
X_1_ X_2_	1	4.53	4.53	2.16	0.20		
X_1_ X_3_	1	0.22	0.22	0.11	0.76		
X_2_ X_3_	1	0.40	0.40	0.19	0.68		
Residual	5	10.47	2.09				
Lack of fit	3	7.48	2.49	1.67	0.40		
Pure error	2	2.99	1.49				
Total	14	817.48					

**Table 4:** Validation of the optimized extraction conditions.

	Predicted Yield of Oxypeucedanin Hydrate (%)	Observed Yield of Oxypeucedanin Hydrate (%)	Predicted Yield of Byakangelicin (%)	Observed Yield of Byakangelicin (%)
Mean	97.99	98.06	98.01	99.52
Standard Deviation		2.37		0.96
Relative Standard Deviation (%)		2.42		0.96

**Table 5:** Recovery of oxypeucedanin hydrate and byakangelicin.

Back-Extraction Solvent	Recovered Amount of Oxypeucedanin Hydrate (μg)	Recovered Amount of Byakangelicin (μg)	Yield of Oxypeucedanin Hydrate (%)	Yield of Byakangelicin (%)
DIW	332.15	300.50	54.46	41.18
0.01 N HCl	517.79	570.98	84.90	78.24
0.01 NaOH	0	0	0	0

**Table 6:** Recovery of oxypeucedanin hydrate and byakangelicin using distinct volumes of 0.01 N HCl.

Volume of 0.01 N HCl (mL)	Recovered Amount of Oxypeucedanin Hydrate (μg)	Recovered Amount of Byakangelicin (μg)	Yield of Oxypeucedanin Hydrate (%)	Yield of Byakangelicin (%)
10	342.22	257.22	56.11	35.24
20	441.64	538.66	72.41	73.81
30	529.42	580.29	86.81	79.51
40	561.06	650.762	91.99	89.17
50	541.69	660.012	89.47	90.44

**Table 7:** Contents of oxypeucedanin hydrate and byakangelicin in the product.

	Recovered Amount (μg)	Yield (%)	Content (%)
Total extract	14,456.80	-	-
Oxypeucedanin hydrate	5347.09	90.17	36.99
Byakangelicin	6523.39	88.51	45.12

**Table 8:** Comparison of the extraction ability of different solvents.

Extraction Solvent	Amount of Oxypeucedanin Hydrate (μg)	Amount of Byakangelicin (μg)	Extraction Yield of Oxypeucedanin Hydrate (%)	Extraction Yield of Byakangelicin (%)
DIW	462.42	625.01	37.90	41.39
50% ethanol	885.39	782.64	72.57	51.83
95% ethanol	946.18	1288.83	77.56	85.35
[Bmim]Tf_2_N	1194.89	1498.55	98.06	99.52

## Sample Availability

Samples of the compounds are available from the authors.
